# A Massively Parallel Sequence Similarity Search for Metagenomic Sequencing Data

**DOI:** 10.3390/ijms18102124

**Published:** 2017-10-11

**Authors:** Masanori Kakuta, Shuji Suzuki, Kazuki Izawa, Takashi Ishida, Yutaka Akiyama

**Affiliations:** 1 Department of Computer Science, Graduate School of Information Science and Engineering, Tokyo Institute of Technology, 2-12-1 W8-76 Ookayama, Meguro-ku, Tokyo 152-8550, Japan; kakuta@bi.cs.titech.ac.jp (M.K.); suzuki@bi.cs.titech.ac.jp (S.S.); ishida@c.titech.ac.jp (T.I.); 2 Education Academy of Computational Life Sciences (ACLS), Tokyo Institute of Technology, 4259 J3-141 Nagatsuta-cho, Midori-ku, Yokohama, Kanagawa 226-8503, Japan; 3 Department of Computer Science, School of Computing, Tokyo Institute of Technology, 2-12-1 W8-76 Ookayama, Meguro-ku, Tokyo 152-8550, Japan; izawa@bi.c.titech.ac.jp

**Keywords:** database search, sequence similarity search, metagenomics, human oral microbiome

## Abstract

Sequence similarity searches have been widely used in the analyses of metagenomic sequencing data. Finding homologous sequences in a reference database enables the estimation of taxonomic and functional characteristics of each query sequence. Because current metagenomic sequencing data consist of a large number of nucleotide sequences, the time required for sequence similarity searches account for a large proportion of the total time. This time-consuming step makes it difficult to perform large-scale analyses. To analyze large-scale metagenomic data, such as those found in the human oral microbiome, we developed GHOST-MP (Genome-wide HOmology Search Tool on Massively Parallel system), a parallel sequence similarity search tool for massively parallel computing systems. This tool uses a fast search algorithm based on suffix arrays of query and database sequences and a hierarchical parallel search to accelerate the large-scale sequence similarity search of metagenomic sequencing data. The parallel computing efficiency and the search speed of this tool were evaluated. GHOST-MP was shown to be scalable over 10,000 CPU (Central Processing Unit) cores, and achieved over 80-fold acceleration compared with mpiBLAST using the same computational resources. We applied this tool to human oral metagenomic data, and the results indicate that the oral cavity, the oral vestibule, and plaque have different characteristics based on the functional gene category.

## 1. Introduction

Most microbes are difficult to isolate and cultivate [1]. The metagenomic approach with direct sequencing of microbial genomes from environmental samples is a culture-independent way to identify uncultured microbes. Metagenomic studies have been conducted in the human body [2,3], soil [4], seawater [5], and air [6], and the identification of novel genes and species have provided us with new information about microbes in various environments. Moreover, metagenomic studies have reported relationships between genes in microbial communities and environmental conditions. For example, Tringe et al. sequenced indoor air microbes and compared overrepresented genes with those from environmental sources such as seawater, soil, and whale fall [6].

In such studies, environmental samples are characterized by the abundance of ortholog groups [2,6,7]. Studying these abundances enables us to uncover the relationships between gene functions and environmental conditions. We can reconstruct possible metabolic pathways within the microbial community in an environment, and compare each metagenomic sample based on its gene functions or functional categories [7]. The reconstruction of metabolic pathways provides information about potential metabolites, possible paths to a specific metabolite, and the structure of a metabolic network in an environment.

To estimate the abundance of ortholog groups in environmental samples, sequence similarity searches have been used to identify ortholog groups of each sequence in the metagenomic data. In metagenomic studies, the query sequences often have no close homologs in database sequences. This necessitates sensitive search methods, such as BLASTX [8], which searches an amino acid sequence database for similarities within the translated nucleotide query sequence.

However, searching with BLASTX requires a long calculation time, making it difficult to perform large-scale analyses (i.e., studies including hundreds of environmental samples). For example, each BLASTX search takes about one minute with a single query of a nucleotide sequence of approximately 100 bases and a reference sequence database such as KEGG GENES [9] or NCBI BLAST nr [10]. Metagenomic data with whole genome sequencing using a massively parallel DNA sequencing technique often consists of tens of millions of short reads. Thus, current metagenomic functional annotations using BLASTX require over 1000 days to process metagenomic sequencing data with a single CPU core.

The Human Microbiome Project (HMP) has already achieved large-scale functional analyses, albeit with a markedly reduced reference database [3,7]. Some 681 sets of metagenomic shotgun sequencing data from 18 human body sites were analyzed with the HMP Unified Metabolic Analysis Network (HUMAnN) [7]. However, in the HUMAnN workflow, the subset of the KEGG GENES database used for reference consisted of amino acid sequences from only 28 species. The size of this subset of data is approximately 1% of the whole database. Although they are faster, similarity searches with reduced databases can affect the accuracy and availability of functional annotations because it is more likely that no similar sequence is found and the function of each query sequence may not be estimated. For example, the human oral microbiome constitutes more than 600 bacterial species [11,12]. For a detailed analysis of taxonomic composition and functional genes in the bacterial community, there are strong demands for using whole databases. However, an analysis using the whole database needs to perform calculation-cost. Therefore, there is a need for high-speed sequence similarity search algorithms and massively parallel computations.

Two approaches have been developed to accelerate sequence similarity searches. The first uses a search algorithm with a sophisticated database index, such as a hash table [13] or a suffix array [14,15]. This method avoids linear searching for alignment candidates used in BLASTX [8], and instead uses the index of the database. This shortens one of the most time-consuming parts of the similarity search, and makes the whole search tens of times faster than the BLASTX algorithm. The second approach employs a parallel search on massively parallel computing systems. This technique is particularly useful for metagenomic data produced by massively parallel DNA sequencing because massively parallel sequencing data consist of many nucleotide sequence fragments, and this approach can search for each fragment in parallel. Ideally, the parallel search approach should reduce the execution time in inverse proportion to the number of computational units. Darling et al. developed mpiBLAST [16], which is a parallel implementation of NCBI BLAST using the Message Passing Interface (MPI). The mpiBLAST software searches in parallel using multiple processes on a distributed memory system with thousands of CPU cores to reduce the search time.

Although both approaches accelerate the similarity search process, the acceleration of only one approach is insufficient for large-scale analyses. We typically require 10,000-fold acceleration compared with a single BLASTX run with one CPU core for the functional annotation of shotgun sequencing data within several hours. Search algorithms with database indexes are not fast enough. An ideal parallel search could achieve 10,000-fold acceleration using 10,000 times the computational resources, but those means are not easily available. Thus, a method that combines the advantages of both approaches is needed.

In this study, we developed a new massively parallel sequence similarity search tool for large-scale metagenomic sequencing data, such as the human oral microbiome. The system consists of a parallel sequence similarity search on a massively parallel distributed memory system, named GHOST-MP. This enables the analysis of large-scale metagenomic data consisting of hundreds of sets of environmental sequencing data. GHOST-MP employs both a fast search algorithm and parallel computation to accelerate similarity searches for metagenomic sequencing. To demonstrate the applicability of GHOST-MP to large-scale metagenomic functional analyses, we first present the search speed and scalability of GHOST-MP on two massively parallel computing systems. We then show the results of large-scale sequence similarity searches of actual metagenomic data. GHOST-MP achieved faster sequence similarity searches than mpiBLAST, enabling large-scale functional analyses to be performed within a short period of time. Then, we performed metagenomic analysis of human oral microbiome based on a fullset of functional gene reference using GHOST-MP. The results indicated that oral cavity, oral vestibule, and plaque have different characteristics.

The GHOST-MP program is implemented in C++, and is available under the BSD (Berkeley Software Distribution) License from http://www.bi.cs.titech.ac.jp/ghostmp/.

## 2. Results and Discussion

### 2.1. Evaluation of Scalability and Search Speed

Before performing the analysis of the human oral microbiome, we evaluated the search speed of GHOST-MP, which was measured on two systems: TSUBAME 2.5 at Tokyo Institute of Technology, and the K computer at RIKEN Advanced Institute for Computational Science, using human oral metagenomic shotgun sequencing data queries and the KEGG GENES amino acid sequence database. The scalabilities were evaluated in weak and strong scaling experiments. In the weak scaling setting, the number of query sequences per CPU core was fixed as the number of cores increased. This scenario evaluates how a large problem can be efficiently dealt with. In the strong scaling setting, the total number of query sequences was fixed to evaluate how fast the method could process the same amount of data. On TSUBAME 2.5, the search speed of mpiBLAST (version 1.6.0) was also measured and compared with that of GHOST-MP using human tongue dorsum metagenomic data (SRS078182). Parts of the query sequences (1,280,000 and 80,000 query sequences for GHOST-MP and mpiBLAST, respectively) were used for evaluation on TSUBAME 2.5 due to limitations in computational resources. mpiBLAST was not evaluated on the K computer because it encountered a bus error on this system. This error could have been caused by unaligned memory access, as the processor in the K computer does not allow such access.

**Figure 1:**
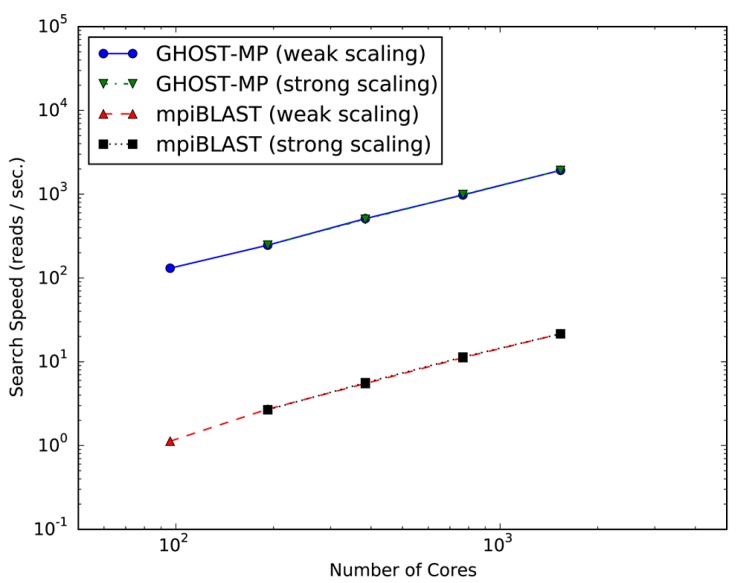
Scalability of GHOST-MP (Genome-wide HOmology Search Tool on Massively Parallel system) and mpiBLAST on TSUBAME 2.5. Strong scaling and weak scaling were measured using 96–1536 CPU cores. In the strong scaling setting at 96 CPU cores, the search speed could not be measured due to computational resource limitations.

Figure 1 plots the search speeds and scalability of GHOST-MP and mpiBLAST on TSUBAME 2.5. Both GHOST-MP and mpiBLAST achieved almost linear scalability, and the search speed of GHOST-MP was 87–115 times faster than that of mpiBLAST. Scalability means that the serial sections of GHOST-MP and mpiBLAST, such as I/O (Input/Output) and scheduling, account for only a small fraction of the computation time compared with the parallelizable sequence similarity search sections at various scales, in which computational resources were efficiently used. The acceleration of GHOST-MP compared with mpiBLAST should arise from the difference between the GHOSTX and BLASTX algorithms. Furthermore, similar accelerations were observed in experiments with a compute node [15].

**Figure 2:**
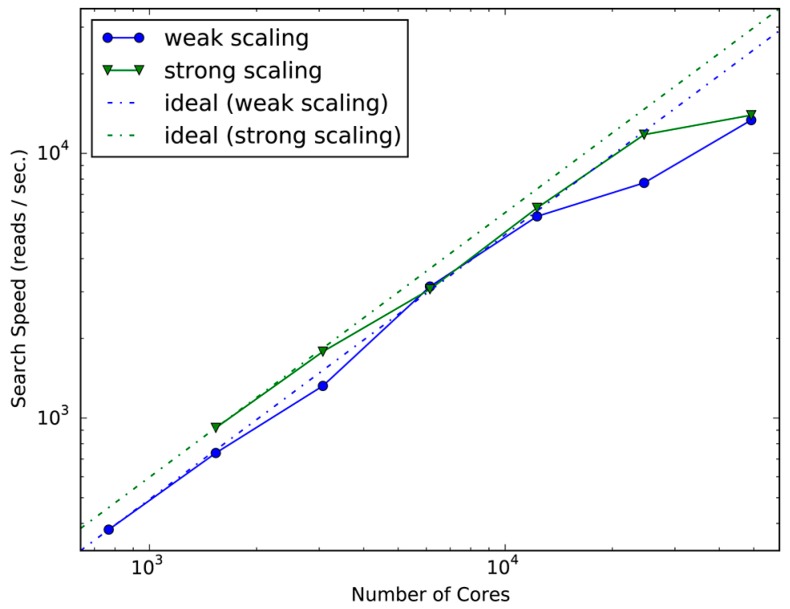
Scalability of GHOST-MP on the K computer. Strong scaling and weak scaling were measured using 768–49,152 CPU cores. The similarity search of the sequencing data with the KEGG GENES amino acid sequence database on the K computer took 183 h (898,231 core hours, which is the product of the number of CPU cores used and the elapsed time). Searches of all sequencing data (except SRS055118) were completed within 2 h (Table S1). The combination of a sophisticated search algorithm with database indexing and a massively parallel search allowed us to achieve this large-scale similarity search within a short period of time.

We further evaluated the scalability of GHOST-MP on the K computer to investigate its performance on a massively parallel computing system. To evaluate the aforementioned scalability, we used 107 samples of buccal mucosa metagenomic data because the K computer has a larger number of CPUs that can carry out a computational process for a larger dataset necessary for evaluation. Generally, it is more difficult to achieve good scalability on larger systems because the master process must communicate with more workers. However, GHOST-MP scaled well up to over 10,000 CPU cores in reference to both criteria (Figure 2). GHOST-MP took 1.73 h to process the whole dataset with 24,576 cores. However, the search speed decreased compared with the ideal speed with 24,576 cores (weak scaling) and 49,152 cores (strong scaling). This decrease in search speed under weak scaling indicates that additional data cannot be efficiently processed, whereas the search speed under strong scaling suggests that no further acceleration can be achieved by increasing computational resources. The performance drop may have been caused by the contention of point-to-point communications between the master and workers. To make the parallel search more scalable for large-scale analyses, it is necessary to reduce the contention. Introducing multiple masters or submasters at the MPI level or implementing collective communication instead of point-to-point communication may address this problem.

### 2.2. Large-Scale Sequence Similarity Search for Metagenomic Sequencing Data

To demonstrate the applicability of GHOST-MP to large-scale functional analysis of metagenomic data, we applied the functional analysis workflow to healthy human oral metagenomic data consisting of 381 samples taken from eight oral sites, with approximately 18 billion sequence reads (Table S2). Through the functional gene analysis pipeline, 109,127,620 reads (0.6% of the total) and 75,363,198 reads (0.4% of the total) were filtered out by a similarity search against the NCBI nr and KEGG GENES databases, respectively. A total of 10,357,599,878 reads (56.0% of the total) were aligned to similar sequences in the KEGG GENES database. The search results are summarized in Table S2. We used the relative abundance of orthologous gene groups in each sample and the results of the workflow to compare metagenomic samples between oral sites. Relationships among metagenomic samples were summarized using principal component analysis (PCA). Orthologous gene groups with relative abundances of less than 0.0001 in all samples were excluded in advance. In other words, the number of orthologous groups decreased from 6881 to 3181. However, the remaining orthologous groups account for most of the total relative abundances, and the sums of the relative abundances are >0.98 in all samples. These relative abundances were transformed into principal components by PCA.

**Figure 3:**
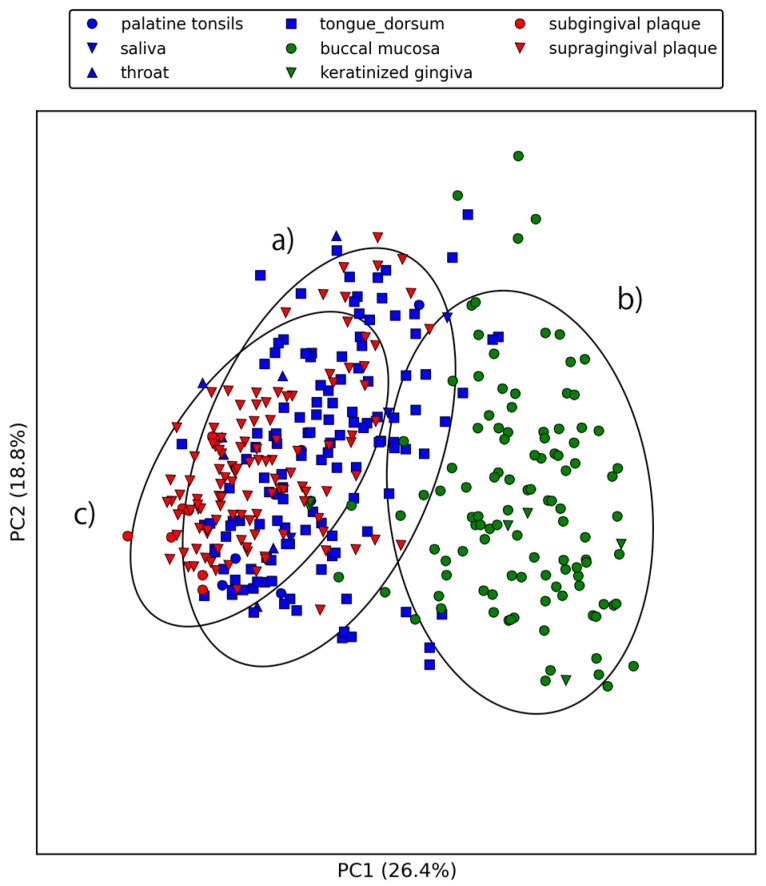
First and second principal components of the relative abundances of orthologous groups. Ellipses represent the covariance (2σ) of the three oral site groups. (**a**) indicates oral cavity (palatine tonsils, saliva, throat, and tongue dorsum), (**b**) indicates oral vestibule (buccal mucosa and keratinized gingiva), and (**c**) indicates plaque (subgingival plaque and supragingival plaque).

**Figure 4:**
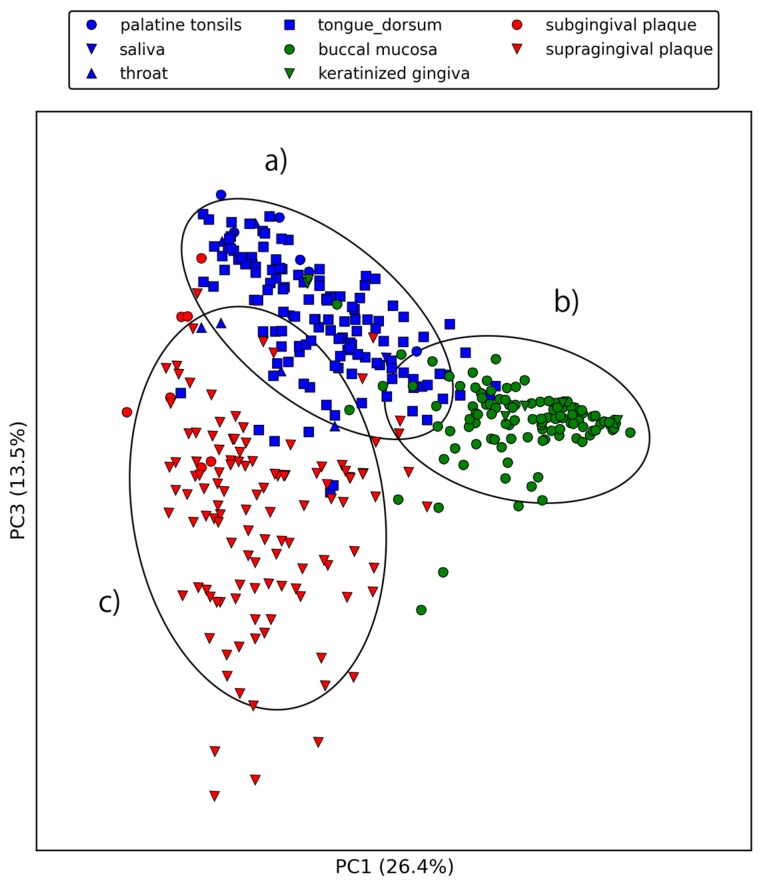
First and third principal components of the relative abundances of orthologous groups. (**a**) represents the oral cavity (palatine tonsils, saliva, throat, and tongue dorsum); (**b**) represents the oral vestibule (buccal mucosa and keratinized gingiva), and (**c**) represents plaque (subgingival plaque and supragingival plaque).

**Figure 5:**
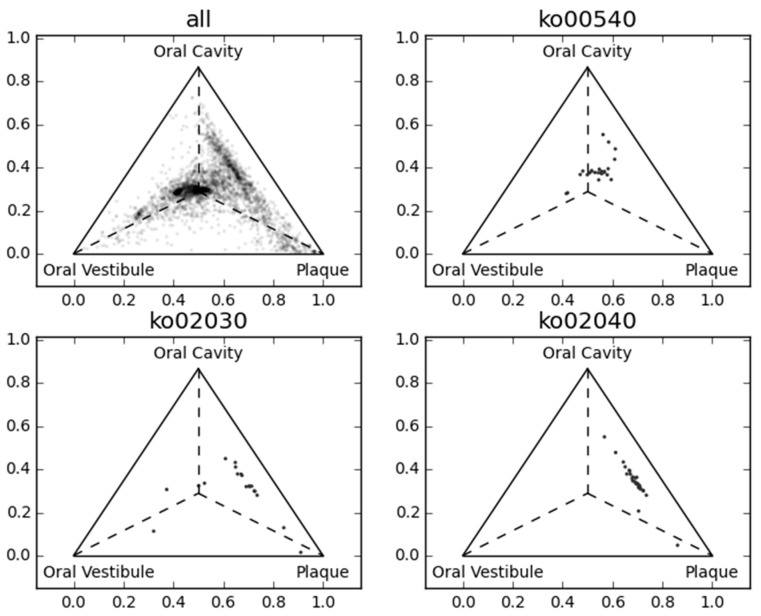
Comparison of the average relative abundance among three oral site groups. Orthologous groups are represented as dots in each ternary plot based on their average relative abundance. The centroid of the triangle represents an equal average relative abundance of the three oral site groups.

Figure 3 and Figure 4 show the first three principal components of oral samples. The three principal components describe the relationships among samples well and account for 58% of the total variance. In particular, samples from the same oral sites tend to make clusters with regard to the first and third principal components, and we can group the eight oral sites into three groups (Figure 4). These groups were composed of (a) the oral cavity; (b) the oral vestibule; and (c) plaque. This result is consistent with a phylogenetic analysis of human oral microbiomes [17]. The average relative abundances of the orthologous groups in these oral site groups were also investigated. There was a large number of orthologous groups that are more or less abundant in specific oral site groups (Figure 5). Some orthologous groups related to specific biological pathways were found to be abundant in specific oral site groups. For example, orthologous groups related to the lipopolysaccharide biosynthesis (PATH: ko00540) are abundant in the oral cavity. This suggests an abundance of Gram-negative bacteria, which have lipopolysaccharide in their outer membrane, in the oral cavity. Almost all orthologous groups related to bacterial chemotaxis (PATH: ko02030) and flagellar assembly (PATH: ko02040) according to the KEGG PATHWAY are abundant in the oral cavity and plaque. Genes related to these pathways are involved in microbial motility, and it has been reported that genes related to microbial motility are over-represented in plaque microbiomes of periodontal disease compared with those of healthy periodontal tissue [18,19,20]. Through this large-scale functional analysis, we have confirmed the applicability of GHOST-MP to current metagenomic shotgun sequencing data.

## 3. Materials and Methods

### 3.1. Sequence Data

Human oral metagenomic sequencing data were downloaded from the HMP Data Analysis and Coordination Center (HMP DACC; http://www.hmpdacc.org). Reads from the human genome, duplicate reads, and low-quality bases were removed from these data by HMP DACC in advance. Identifiers and details of human oral samples data are listed in Table S2.

The KEGG GENES database [8] (released July 2012) was used for reference sequences. This database contains 8,578,853 amino acid sequences.

### 3.2. Functional Gene Analysis Pipeline

The functional analysis pipeline mainly consists of four steps. The first step trims away the low-quality tails from the reads. The entire read is filtered out if the remaining sequence is shorter than 60. The second step filters out the reads derived from Eukaryotes. The reads are considered as derivation from Eukaryotes if the top hit of the sequence similarity search against NCBI nr database (accessed July 2012) is the sequence of Eukaryotes. The third step maps the read sequences to the annotated sequences in the KEGG GENES amino acid sequence database (released in July 2012). The second step and the third component perform sequence similarity searches using GHOST-MP with the PAM 30 substitution matrix, with the gap opening penalty of −9 and the gap extension penalty of −1. These steps account for most of the computation time in this workflow. The search results are considered as hits if the alignment score and sequence identity are above 40% and 70%, respectively. The fourth one calculates the relative abundances of orthologous groups (KEGG Orthology entries) in the data. The number of hits of each gene in an orthologous group is summed up with normalization using its gene length and the number of hits of universal single-copy genes [21].

### 3.3. Computing Environments

The TSUBAME 2.5 supercomputer consists of 1408 thin compute nodes. Each compute node has two Intel Xeon X5670 processors (2.93 GHz, six cores) and 54 GB of main memory. The nodes are interconnected with a full bisection-bandwidth fat-tree network. Each compute node has three NVIDIA Tesla K20X GPU accelerators, but the accelerators were not used in this study. The K computer consists of 82,944 compute nodes. Each compute node has a SPARC64 VIIIfx processor (2.0 GHz, eight cores) and 16 GB of main memory, and is connected to a six-dimensional mesh/torus network. We used up to 1536 CPU cores (128 nodes) and 49,152 CPU cores (6144 nodes) to measure the scalability of GHOST-MP on TSUBAME 2.5 and the K computer, respectively.

### 3.4. Sequence Similarity Search with Indexes Based on Suffix Arrays

GHOST-MP uses the GHOSTX [15] search algorithm for sequence similarity search. The GHOSTX program achieves more than 100-fold acceleration over the BLASTX algorithm, albeit with a slight decrease in search sensitivity. Briefly, the algorithm uses suffix arrays [22] as an index to accelerate the search for alignment candidates. The suffix array data structure is a sorted array of indexes of all the suffixes of a string in lexicographical order. The suffix array can be used with a binary search to find all suffixes matching the query string, and is a data structure widely used in biological sequence searches [23]. Binary searches on the suffix array produce efficient enumeration of all intervals in the suffix array that start with each letter representing an amino acid type. We can recursively apply the same procedure for subsequent letters to narrow down the intervals. Moreover, the GHOSTX algorithm uses an additional data structure (an array of ranges of the same fixed length prefixes of the suffixes in the suffix array) to avoid several initial steps in the binary search. These data structures make it possible to obtain intervals as alignment candidates by filtering out dissimilar intervals in terms of a substitution matrix score.

The main search algorithm consists of the following steps: (1) search for alignment candidates with the suffix array; (2) perform ungapped extension of the candidates; (3) filter out overlapping candidates; and (4) perform gapped extension of the candidates. Query nucleotide sequences are treated as amino acid sequences throughout the search procedure, with translations over six possible reading frames for a sensitive search with an amino acid substitution matrix.

### 3.5. Hierarchical Parallelization of the Sequence Similarity Search with Data Parallelism

GHOST-MP adopts a two-level hierarchical parallelization. The sequence similarity search is parallelized by MPI at the inter-node level and by OpenMP at the intra-node level. The original GHOSTX algorithm only provides a parallel similarity search with OpenMP. Thus, we could not execute GHOSTX on distributed-memory systems (inter-node), which account for a large portion of current supercomputers in use, because OpenMP only provides parallelization on shared-memory systems (intra-node). Thus, GHOSTX could not take advantage of the large computational power of supercomputers. The hierarchical parallelization has two advantages compared with MPI-only parallelization, described as follows.

(1) Hierarchical parallelization largely reduces the memory use of worker processes because it enables the sharing of common data in intra-processes, such as database sequences. The size of database sequences and their index often exceed the memory size in massively parallel environments. Index size is the product of the length of the concatenated database sequence and the size of the index pointing to the corresponding position in the concatenated database sequence. For example, when KEGG GENES (3.5 GB, released July 2012) is used as an amino acid sequence database, the total size of the database sequence and its suffix array with auxiliary data is approximately 20 GB. If we use MPI for both inter- and intra-node parallelization, each individual process, even those within the same computing node, has to store the same database. In current massively parallel computing systems, nodes rarely have sufficient memory to store multiple copies of the database and its index. To reduce memory use, it is possible to split the database by assigning different partitions to each intra-node process. However, searching by this approach is inefficient for two reasons. First, splitting the database requires an additional merging step to combine the most similar hits for the same query sequence. Second, searching for alignment candidates with a split database requires more CPU time than searching with an unsplit database because the search time for alignment candidates with a suffix array is proportional to the logarithm of the database size.
(2) Hierarchical parallelization can also lead to scalable parallel searching. Since the communication between the master and the workers involves MPI point-to-point communication, parallel searches with a smaller MPI process reduce the number of communications sent from the workers to the master compared with searches in nonhierarchical parallelization (MPI-only parallelization).

**Figure 6:**
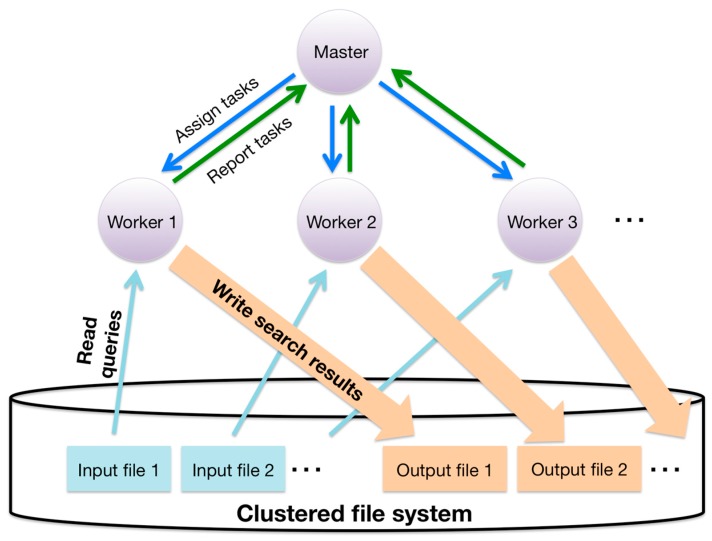
Schematic of task distribution and file I/O (Input/Output) in GHOST-MP.

Details of this two-level hierarchical parallelization of GHOST-MP are as follows. At the inter-node level, GHOST-MP adopts a master–worker model. Communication between the master process and the worker process is implemented with MPI. Firstly, query sequences are split into the same number of chunks as the number of worker processes. The master process assigns a sequence chunk to each worker process as a task. At the intra-node level, similarity searches are parallelized with OpenMP. Query sequences in a chunk are subdivided into more specific tasks. These subdivided tasks are put into a queue, and each OpenMP thread sequentially dequeues a task from the queue using a lock. Finally, each worker process writes search results to a clustered file system and reports results to the master process (Figure 6).

## 4. Conclusions

We have developed GHOST-MP, a massively parallel sequence similarity search tool for metagenomic data to allow for large-scale analyses of metagenomic data. GHOST-MP uses a search algorithm with suffix arrays, and its parallel similarity search procedure is implemented using a two-level hierarchical model. GHOST-MP achieved over 80-fold acceleration compared with mpiBLAST, and exhibited almost a linear increase in speed with an increase in the number of CPU cores on TSUBAME 2.5. GHOST-MP also scaled well to over 10,000 CPU cores on the K computer. The fast search ability of GHOST-MP enabled us to perform a large-scale sequence similarity search of 381 human oral microbiome metagenomic sequencing data (18 billion reads) against the whole database of KEGG GENES (8.6 million amino acid sequences) on a massively parallel computing system. This massive data analysis indicated characteristics of functional gene in the microbial community of three human oral parts.

## Supplementary Materials

Supplementary materials can be found at www.mdpi.com/1422-0067/18/10/2124/s1.
